# Unusual Chronotropic Incompetence in a Young Patient: Mitigating the Fear of Fatal Bradyarrhythmia When Antipsychotics Are Deemed Necessary

**DOI:** 10.7759/cureus.90033

**Published:** 2025-08-13

**Authors:** Prutha V Patel, Yash K Desai, Minaal Khan, Zaid A Naseer, Michael Marney, Ahmed M Exeer, Gul P Osmani, Mahmood Sulliman, Partam Manalai

**Affiliations:** 1 Internal Medicine, Edward Via College of Osteopathic Medicine, Blacksburg, USA; 2 Psychiatry and Behavioral Sciences, Liberty University College of Osteopathic Medicine, Lynchburg, USA; 3 Internal Medicine, Mary Washington Healthcare, Fredericksburg, USA; 4 Psychiatry, Intuitive Insight Inc., McLean, USA; 5 Psychiatry, Mary Washington Healthcare, Fredericksburg, USA

**Keywords:** antipsychotic medication, bradyarrhythmia, cardiac complications, chronotropic incompetence, psychopharmacology

## Abstract

Severe chronotropic insufficiency, while uncommon in psychiatric populations, presents unique challenges in patients with psychotic disorders because many antipsychotics can adversely affect cardiac rhythm by exacerbating bradycardia or prolonging the QT interval. This case report describes a young male with a complex psychiatric history, severe substance use disorder, and coexisting cardiac abnormalities, who presented with profound sinus bradycardia and a prolonged QT interval. The patient's psychiatric symptoms were compounded by medication nonadherence and illicit drug use. The patient had a history of profound sinus bradycardia (heart rates in the 20s to 30s) with a mean heart rate of 41 beats/min (SD = 4), yet remained asymptomatic from a cardiovascular perspective. Despite the presence of bradycardia and a prolonged QT interval (mean QT = 495 ms (66), QTcB = 427 (30) (Bazett), and QTcF = 448 (32) (Fridericia)), he was treated with oral olanzapine and valproate. His psychiatric symptoms improved significantly, with no exacerbation of bradycardia or QTc prolongation noted during treatment.

This is a rare case of a hemodynamically stable young man treated with second-generation antipsychotics, without any notable effect on the length of the QT interval. QT prolongation is a side effect of many antipsychotics, and bradycardia tends to further prolong the QT interval, increasing the risk of adverse cardiac outcomes, such as torsades de pointes or sudden cardiac death, for patients taking antipsychotics. An interprofessional, interdisciplinary, and cohesive team can reduce the cardiac risk to the patient while effectively treating the underlying psychiatric disorders. Nonetheless, it is essential for psychiatrists to have a solid understanding of psychotropic medications and cardiac rhythms, particularly because many free-standing psychiatric facilities do not have direct access to a cardiology consult team. In this report, the authors will attempt to provide a structured framework for psychiatrists managing patients with bradycardia who require antipsychotic therapy.

## Introduction

Sinus bradycardia, a condition characterized by an abnormally slow heart rate, poses unique clinical challenges, particularly when present in young patients requiring psychotropic medications [1,2]. The interplay between severe bradycardia and the use of antipsychotics, a class of drugs often associated with corrected QT interval (QTc) prolongation and arrhythmogenic potential, necessitates a strategic approach to management [3]. Misinterpretation of QTc measurements in the context of bradycardia can further complicate decision-making, leading to either unnecessary avoidance of essential psychiatric medications or undue risks of cardiac events [4].

This case report discusses a rare presentation of asymptomatic sinus bradycardia in a young adult male, with heart rates as low as 20-30 beats per minute and a prolonged QT interval on admission that corrected to normal upon adjustment. The case highlights the importance of a multidisciplinary management strategy, contextualizing QT interval interpretation, individualized antipsychotic selection, and ongoing cardiac monitoring to mitigate adverse cardiovascular risks. While single-patient findings are inherently limited in generalizability, this case highlights practical considerations applicable in similar complex presentations. By examining this patient's course, the authors highlight practical considerations and propose a clinical framework that can guide psychiatrists in safely managing antipsychotic therapy for patients with significant bradycardia.

## Case presentation

A 33-year-old male with a history of bipolar I disorder, along with methamphetamine, cocaine, as well as other substance use disorders, presented with worsening depression, suicidal ideation, hallucinations, and delusions. His psychiatric symptoms were compounded by social stressors, medication nonadherence, and reported illicit substance use. On admission, the patient demonstrated severe psychomotor withdrawal, emotional lability, and limited cooperation with medical evaluations or treatment planning. Initial medical evaluation revealed no acute medical comorbidities, and routine laboratory workup, including complete blood count, comprehensive metabolic panel, and liver function tests, was within normal limits.

The patient met diagnostic criteria for bipolar I disorder, with the most recent episode being depressed with psychotic features. The patient also met diagnostic criteria for post-traumatic stress disorder (PTSD); severe cannabis, cocaine, and methamphetamine use disorders; and a prior history of other substance use disorders, including opioids. Initial treatment efforts were complicated by his resistance to engage, but over the course of hospitalization, he became more selectively cooperative and participated actively in discussions about his care, although he declined to complete several electrocardiogram (ECG) orders. The patient was ultimately stabilized on valproic acid 500 mg three times daily (total 1500 mg/day) and olanzapine 5 mg nightly. Urine drug screens across multiple admissions consistently confirmed ongoing multi-substance use, including cannabis, cocaine, methamphetamine, alcohol, and occasional opiate use.

Cardiac findings

**Figure 1:**
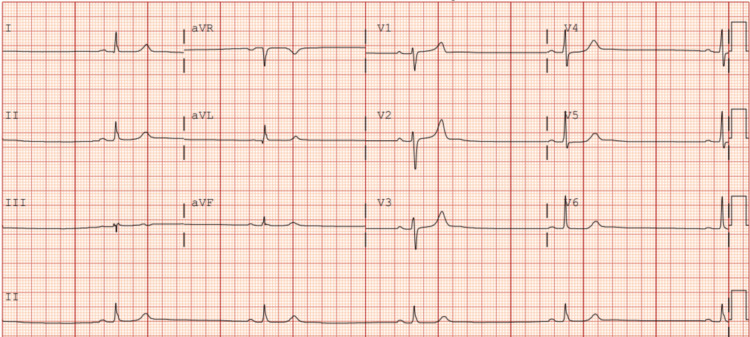
Patient’s ECG reading shows sinus bradycardia at 29 bpm. The ECG reading also indicates a QT interval of 557 ms and a QTcB of 388 ms.

Concurrent with his psychiatric symptoms, the patient exhibited profound sinus bradycardia, with historic resting heart rates as low as the 20s to 30s (Figure 1). Despite the severity of his bradycardia, he remained asymptomatic from a cardiovascular standpoint, with no episodes of syncope or significant hemodynamic compromise. An ECG revealed a QT interval of 557 ms, which corrected to 388 ms using the Bazett formula (Figure 1); this falls within the reference range for QTc (350-450 ms in males). This degree of correction, while within standard calculation methods, may reflect the known limitations of Bazett’s formula in the setting of extreme bradycardia. Therefore, both Bazett (QTcB) and Fridericia (QTcF) corrected QT values were reported to provide a more comprehensive interpretation.

The patient's history included a treadmill stress test in 2017, which demonstrated chronotropic incompetence, with an inadequate heart rate response to exercise. At the time, the baseline ECG showed sinus bradycardia with a heart rate of 42 beats per minute (bpm). Blood pressure was 124/84 mm Hg, and oxygen saturation was 98%. There was no new ST-segment depression. During stress, the patient developed frequent premature atrial contractions (PACs) and was unable to achieve the target heart rate due to dyspnea, leg fatigue, and verbal combativeness, which was the most likely reason for not completing the test. The blood pressure response to exercise was normal, reaching 163/74 at peak stress, with oxygen saturation remaining at 98%. The peak heart rate was 80 bpm, but heart rate recovery was abnormal. Metabolic equivalent of task (MET) estimates the amount of energy used during exercise. One unit MET (1 MET) is approximately 3.5 (ml/kg/min). The double product is calculated as the heart rate multiplied by the systolic blood pressure. The Duke Treadmill Score refers to a clinical score used to assess a patient's risk of cardiovascular events based on their treadmill exercise test results. The functional capacity was poor, with an estimated metabolic workload of 7.0 METs equivalent to moderate exercise and a peak double product of 12008 mmHg/min, indicating a relatively high cardiovascular workload. The Duke Treadmill Score was 6.0, indicative of low risk for cardiovascular events. Due to a lack of compliance with recommendations, there were significant challenges with follow-up. Nonetheless, the first echocardiogram (date of completion: 7/3/2021) was benign. The sizes of the left and right atria were normal. The left ventricle was normal in size with normal wall thickness, and its systolic function was normal, with an ejection fraction of 55-60%. The left ventricular wall motion was also normal. The right ventricle was of normal size, and its systolic function was normal. There was trace to mild (1+) mitral regurgitation and trace to mild tricuspid regurgitation. No pericardial effusion was present. The repeat echocardiogram findings were also reassuring (6/8/2024) when he presented again to this hospital's emergency room. The left ventricle was of normal size with normal systolic function and an ejection fraction of 60-65%. Its wall motion and thickness were also normal. The right ventricle was normal in size and function. There were no hemodynamically significant valvular abnormalities, with no pericardial or pleural effusions present. The diastolic flow pattern was normal.

Seventeen ECGs were completed between 7/11/2017 and 11/25/2024, though the patient inconsistently followed through with testing and did not complete several ordered ECGs. The analysis revealed the following average values for key ECG intervals: the RR interval had a mean of 153.4 ms with a standard deviation (SD) of 23.5 ms, the QRS duration interval averaged 91.8 ms (SD = 7.5 ms), and the QT interval had a mean of 510.1 ms (SD = 59.2 ms). Mean QT interval was 495.4, with a 95% confidence interval (CI) of 462.865-527.935. The corrected QT intervals were also calculated, with the QTcB having a mean of 436.1 ms (SD = 25.5 ms) and the QTcF showing a mean of 451.4 ms (SD = 26.6 ms).

Variability between successive heartbeats (VSHB) is defined as the difference between two successive heart rates. There was no significant difference when the patient presented with a urine drug screen (UDS) positive for cocaine alone, cocaine and methamphetamine, or methamphetamine alone, compared to when the UDS was negative for both drugs (F-ratio = 2.65, p = 0.065). When ECG findings with positive UDS results for cocaine and methamphetamine (either/both positive) were clustered together and compared to ECG findings for all admissions with negative UDS for both drugs, there was no difference in VSHB (t = -1.45, p = 0.075). Heart rate variability (HRV) for those admissions with positive UDS for cocaine and/or methamphetamine was 271.4 ms, while HRV for UDS negative admissions was 272.3 ms, compared to the normal range of 50-100 ms. The QT variability index (QTVI) was 12.9, which is markedly elevated and consistent with significant autonomic dysregulation. This can increase arrhythmogenic risk, especially in the setting of bradycardia and psychotropic medications. Troponin levels had been normal (<0.002) on every admission. All other laboratory workups, including complete blood count, comprehensive metabolic panel, thyroid-stimulating hormone, lipid panel, and urine drug screen, were unremarkable. Cardiology recommended outpatient follow-up, possibly with mobile cardiac outpatient telemetry (MCOT) or a cardiac event monitor; however, the patient did not follow up after discharge. Since bradycardia was persistent without any symptoms, conservative outpatient cardiac care was recommended, and the patient was scheduled to follow up with cardiology.

Management and course

The patient carried a principal diagnosis of bipolar I disorder; therefore, the patient was started on valproic acid 500 mg two times daily and dose-adjusted to 500 mg three times daily after an initial level of 48 mcg/mL, which was below the therapeutic range. Considering he had significant psychotic symptoms upon presentation as well as profound depressive symptoms, an antipsychotic was deemed necessary. Aripiprazole is less likely to increase the QT interval and thus was initially recommended; however, the patient had a negative prior experience with it and refused it [5]. The choice of olanzapine 5 mg at bedtime was based on the patient’s unwillingness to accept treatment with aripiprazole and its relatively favorable profile in terms of QTc prolongation risk, though ongoing cardiac monitoring was deemed essential due to the patient’s bradycardia [6]. There was no observed change in heart rate on routine vital sign monitoring, though a repeat ECG was declined. On day eight of admission, the patient's symptoms significantly improved, and the patient requested to be discharged.

Throughout hospitalization, the patient’s psychiatric symptoms improved notably. His hallucinations and delusions diminished, his mood stabilized, and his suicidal ideation resolved. Despite his bradycardia, the decision to continue antipsychotic therapy was ethically justified, as the potential risks of untreated psychosis, including the high likelihood of self-harm, outweighed the cardiovascular risks. This approach aligned with a harm-reduction strategy, emphasizing the necessity of treatment while minimizing potential adverse effects [7]. The cardiology consultation team recommended a conservative approach with outpatient referral to cardiology. Notably, psychiatric symptoms improved during inpatient treatment with no ECG-documented deterioration in cardiac function during this phase.

## Discussion

This patient’s history of polysubstance use, including cocaine and methamphetamine, may have contributed to bradycardia [7]. These two sympathomimetics are typically associated with tachyarrhythmias but can, in chronic users, lead to autonomic dysfunction that results in bradycardia [7]. Persistent cocaine use has been reported in the literature to be associated with sinus bradycardia, and sinus bradycardia can be one clinical indication of chronic cocaine use [7,8]. One specific study reported reduced heart rate in cocaine users, where the severity of bradycardia was associated with the length of cocaine use [9]. Compared to cocaine users without bradycardia, the study found that those with bradycardia exhibited an increased rate of early cardiac repolarization, with the severity of bradycardia positively correlating with the extent of early repolarization. Other studies have indicated that methamphetamine use is associated with a 27% increase in sudden cardiac death [8]. Although the mechanism at the molecular level remains elusive, methamphetamine predisposes users to ventricular arrhythmias [7]. Although methamphetamine- or cocaine-induced paradoxical chronotropic insufficiency could not be entirely ruled out, the patient’s cardiac findings during a UDS negative for both substances suggest alternative etiologies, such as intrinsic sinus node dysfunction, medication effects, or autonomic imbalance.

Although the cardiology team was consulted initially and remained engaged, due to the chronic, asymptomatic nature of the patient’s bradycardia and previous comprehensive evaluations, the recommendation remained conservative treatment, and the patient was not a candidate for a cardiac pacemaker. The patient’s unwillingness to follow up with cardiology upon discharge reflects the limitations common in community stand-alone psychiatric hospitals. To enhance care quality, freestanding psychiatric facilities could employ a number of strategies; for example, establishing telemedicine links with a cardiology service for real-time consultations. Another possibility is to develop standardized protocols for cardiac risk monitoring and management, including referral pathways to outpatient cardiology services. And finally, advocating for integration of internists or cardiologists into inpatient psychiatric care teams, particularly for high-risk cases, should be a priority.

For patients with chronic, asymptomatic bradycardia like this patient, recommendations are presented in Table 1.

**Table 1:** Recommendations for patients with chronic, asymptomatic bradycardia.

Recommendation	Details
Regular ECG monitoring	Track QTc and bradyarrhythmia trends [1].
Medication avoidance	Avoid medications with known bradycardic or QT-prolonging effects when alternatives exist [1].
Patient education	Educate patients on recognizing concerning symptoms, such as syncope or dizziness, and emphasize the importance of adherence to follow-up care [1].

Accurate assessment of QT intervals is important in managing patients with bradycardia. The raw QT interval may appear falsely prolonged in slow heart rates, leading to overestimated risks. Using correction formulas, such as Bazett’s (QTc = QT/√RR), can help adjust QT measurements to account for heart rate variations [4]. In this case, the QTc (388 ms) fell within the normal range, providing reassurance that antipsychotic therapy could proceed safely. For context, QTc is typically considered normal when <450 ms in males and <470 ms in females [3]. QTVI values are generally ≤0; elevated values such as this patient’s have been associated with autonomic dysregulation and heightened arrhythmic risk [2]. However, clinicians must interpret QTc values cautiously, considering factors like electrolyte imbalances, drug interactions, and underlying cardiac conditions [10]. The Bazett formula was employed in this case; however, it is well-documented to overcorrect QT intervals in the presence of bradycardia. Alternative correction methods such as the Framingham formula (QT + 0.154 × [1 − RR]) and Hodges formula (QT + 0.00175 × [heart rate − 60]) may provide more accurate QTc calculations in patients with slow heart rates. Applying these corrections could refine risk stratification when interpreting QTc values in similar patients [4].

Antipsychotics influence cardiac function primarily through their effects on ion channels, particularly the delayed rectifier potassium (K+) current (IKr) channel, which governs cardiac repolarization [10]. First-generation antipsychotics (FGAs) such as haloperidol and thioridazine are strongly associated with QT prolongation and subsequent development of torsades de pointes [3]. Thioridazine carries a black-box warning for QT prolongation and is generally avoided in high-risk cardiac patients [2]. More broadly, its use has fallen out of favor due to a poor side effect profile.

While evidence regarding second-generation antipsychotics (SGAs) in the specific context of bradycardia is limited, several agents have been studied more extensively for their impact on QTc intervals (Table 2). Aripiprazole is considered one of the safest SGAs regarding cardiac risk due to minimal QTc prolongation [5]. Quetiapine, though associated with orthostatic hypotension, has a milder impact on QTc than other SGAs, while risperidone necessitates closer monitoring due to dose-dependent QTc effects [11,12]. Further studies are needed to directly evaluate these agents' safety profiles in patients with bradycardia specifically. Table 2 presents a selective list of commonly used SGAs with relevant cardiac considerations; this list is not exhaustive.

**Table 2:** Cardiac considerations for second-generation antipsychotics (SGAs). QTc: corrected QT.

SGA	Cardiac considerations
Aripiprazole	Minimal QT prolongation, safe option for patients with baseline cardiac risks [5].
Risperidone	Associated with dose-dependent QT effects; ECG monitoring is advised during initiation and titration [11].
Quetiapine	Known for orthostatic hypotension and modest QT prolongation; lower doses are recommended in bradycardic patients [12].
Clozapine	It can cause tachycardia and myocarditis but has a minimal effect on QT intervals. Regular cardiac monitoring is crucial, especially during initiation [13].
Ziprasidone	Poses a higher risk of QT prolongation; baseline ECG and follow-up are essential [14].
Lurasidone	Low risk of QTc prolongation; had one of the smallest QTc effects in a large-scale meta-analysis of 15 antipsychotics [15].

The influence of the route of administration on antipsychotic-induced cardiotoxicity, particularly in patients with pre-existing cardiac conditions, is not definitively established in the literature. Instead, studies focus primarily on the risks associated with intravenous (IV) and intramuscular (IM) formulations. IV haloperidol has been strongly associated with QTc prolongation and heightened risk of torsades de pointes, particularly in critically ill populations, as demonstrated by its effects on QT interval dispersion [16,17]. Similarly, high doses of IM antipsychotics, such as haloperidol or ziprasidone, have been shown to acutely prolong the QTc interval, necessitating close monitoring in patients with existing cardiovascular risks [18]. Reports of sudden deaths following IM administration further emphasize the potential for acute adverse effects under certain conditions [19]. However, these risks are not universally applicable to all antipsychotics or all settings, and they often depend on dosage, route, frequency, and the patient’s baseline health.

Given the lack of direct comparative studies between routes, the choice of administration in this patient focused on balancing the urgency of symptom control with cardiovascular safety. Oral olanzapine was selected based on the patient’s previous therapeutic response and relative cardiovascular stability, though more robust data comparing long-term risks between oral (PO), intramuscular (IM), and intravenous (IV) formulations are needed to guide future management. The psychiatry team ultimately made the decision to initiate oral olanzapine after confirming with cardiology that no contraindications were identified. While current evidence advises caution with rapid-acting routes, ongoing monitoring and individualized treatment remain paramount for patients with significant cardiac comorbidities [18,19].

The patient tolerated oral olanzapine without exacerbation of bradycardia, and no QTc prolongation suggestive of heightened arrhythmic risk was observed. This suggests that SGAs can be used cautiously in bradycardic patients when the benefits outweigh the risks. Initiating treatment at the lowest effective dose is advisable to further reduce the risk of QTc prolongation, especially in patients with underlying conduction abnormalities. In general, psychiatrists should prioritize medications with minimal QTc effects and consult cardiology to better understand baseline cardiac risks and ensure an appropriate monitoring plan.

Psychiatrists managing antipsychotic therapy in patients with bradycardia or QT abnormalities can adhere to the following recommendations presented in Table 3.

**Table 3:** Recommendations for psychiatrists managing antipsychotic therapy in patients with bradycardia or QT abnormalities. QTc: corrected QT.

Recommendation	Details
Comprehensive baseline evaluation	Conduct an ECG, electrolyte panels, and a detailed cardiac history before starting antipsychotic therapy [20].
Medication selection	Choose antipsychotics with lower risks of QT prolongation (e.g., aripiprazole and lurasidone). Avoid high-risk agents like ziprasidone or thioridazine unless no alternatives exist.
Collaborative care	Involve cardiologists for patients with significant bradycardia (<40 bpm) or prolonged QTc (>450 ms in males and >470 in females) [2]. Recommendations for stress tests or electrophysiology evaluations can provide additional reassurance.
Ongoing monitoring	Repeat ECGs after medication initiation and dose changes. Consider Holter or event monitoring in patients with borderline QTc or symptomatic bradycardia.
Risk mitigation strategies	Address modifiable factors such as electrolyte disturbances or polysubstance use. Titrate medications, using the lowest effective dose. Review the full medication list for other QT-prolonging agents and discontinue or substitute when possible.
Clinical decision-making framework	QTc <450 ms (males) or <470 ms (females) is generally considered low-risk and generally safe to initiate most antipsychotics. QTc >500 ms universally indicates elevated risk.

While there is limited evidence on the long-term effects of SGAs or FGAs on bradycardia, the cumulative autonomic effects of antipsychotics may pose risks [18]. Monitoring heart rate trends and QTc intervals periodically is advisable for long-term safety. Research exploring these effects is needed to provide clearer guidance for clinicians managing bradycardic patients on chronic antipsychotic therapy.

This case is limited by the patient’s intermittent cooperation with care, including refusal of post-treatment ECG and outpatient cardiology follow-up. These factors constrain our ability to draw longitudinal conclusions regarding the cardiac safety of olanzapine in this patient. However, the observed clinical course remains informative for management strategies in similarly constrained settings.

This case highlights the challenges of managing severe psychiatric illness in patients with significant cardiac comorbidities, particularly in resource-limited settings where cardiology support may be limited. It highlights the need for tailored treatment plans that balance psychiatric stabilization with cardiac safety and ongoing monitoring for adverse outcomes. Ethical justification for antipsychotic use in severe bradycardia hinges on the principle of proportionality, in which untreated psychosis poses a significant risk to the patient and others. The decision to use olanzapine reflects a careful risk-benefit analysis, where potential cardiac risks were mitigated through diligent monitoring, and the psychiatric improvement significantly enhanced the patient’s quality of life.

## Conclusions

This case highlights the challenges of managing psychiatric stabilization in patients with significant cardiac comorbidities, particularly in resource-limited settings. Throughout inpatient treatment, the patient’s QTc remained stable, and no adverse cardiac events were observed following olanzapine initiation, suggesting the feasibility of cautious SGA use in bradycardic patients. The use of oral olanzapine in this patient demonstrated the feasibility of safely treating severe psychosis and mood symptoms, despite coexisting sinus bradycardia and an initially prolonged QT interval that corrected within normal limits. A tailored, multidisciplinary approach was critical, balancing psychiatric and cardiac risks through careful monitoring and harm-reduction strategies. The ethical justification for proceeding with treatment rested on the principle of proportionality, where the benefits of psychiatric stabilization outweighed the potential cardiac risks. This case reinforces the need for ongoing collaboration between psychiatry and cardiology to optimize patient outcomes and calls for further research into the cardiac safety profiles of psychotropic medications in vulnerable populations.
